# Is Avian Malaria Playing a Role in Native Bird Declines in New Zealand? Testing Hypotheses along an Elevational Gradient

**DOI:** 10.1371/journal.pone.0165918

**Published:** 2016-11-01

**Authors:** Chris N. Niebuhr, Robert Poulin, Daniel M. Tompkins

**Affiliations:** 1 Department of Zoology, University of Otago, Dunedin, New Zealand; 2 Landcare Research, Dunedin, New Zealand; King Abdullah University of Science and Technology, SAUDI ARABIA

## Abstract

The mosquito-borne disease avian malaria (*Plasmodium* spp.) has impacted both captive populations and wild individuals of native New Zealand bird species. However, whether or not it is a cause of concern to their wild populations is still unclear. In Hawaii, the disease has been a major factor in the population declines of some native forest bird species, often limiting their elevational distribution due to an inverse relationship between force of infection and elevation. While studies have investigated latitudinal patterns of infection in New Zealand, elevational patterns are unexplored. To address this, a survey was conducted in Nelson Lakes National Park, a site experiencing native bird declines in which disease has been suggested as playing a role, to investigate whether there is a similar inverse relationship in New Zealand. Results from blood samples (n = 436) collected over three seasons across a broad elevational range (650–1400 m) support there being such a relationship. In addition, an overall higher prevalence in non-native (14.1%) versus native birds (1.7%) may indicate differential impacts on these two groups, while particularly high prevalence in non-native *Turdus* spp. supports previous suggestions that they are key reservoir hosts for the disease. Overall, these findings add weight to the hypothesis that avian malaria is playing a role in ongoing declines of native New Zealand birds.

## Introduction

Emerging infectious diseases, defined as disease-causing agents that rapidly increase in geographic range, host range, or prevalence, pose a serious threat to the conservation of global diversity [1]. Often it is native wildlife species that are threatened by non-native parasites they have not evolved with or adapted to [2,3]. Avian malaria is one such disease that may be of concern to the New Zealand avifauna, considered to be the most extinction-prone in the world [2,4].

Avian malaria is a vector-borne disease caused by protozoan parasites of the genus *Plasmodium* [5]. Avian *Plasmodium* spp. are cosmopolitan in range (except Antarctica; [6]) and can infect a broad range of bird hosts causing a range of impacts [7,8]. Infection by malaria parasites consists of two phases, acute and chronic. The acute stage occurs immediately after infection, followed by the chronic stage with some seasonal relapses [9]. The acute phase is characterised by a high level of parasitemia and hosts can show clinical signs such as lethargy, reduced weight gain, and sometimes death [5,10]. Individuals experiencing acute illness may have difficulty foraging or escaping predators [11]. During the chronic phase, hosts experience a low level of parasitemia. These chronic infections can last for years, and can have a mild impact on individual fitness [12].

The most dramatic impact of avian malaria documented resulted from the arrival of *Plasmodium relictum* to Hawaii in the early 20th century [13]. In Hawaii, avian malaria is vectored by the non-native mosquito *Culex quinquefasciatus* and is considered a major factor in the population declines and restricted distribution of native forest bird species [14]. The transmission dynamics of avian malaria varies greatly across elevational gradients in Hawaiian forests, with patterns showing high malaria transmission at low elevations, intermittent transmission at middle elevations, and little to no transmission at high elevations, in direct correlation with mosquito density [9,14].

The presence of *Plasmodium* parasites has been confirmed in native New Zealand avifauna and has been linked to mortality in multiple species [15–17]. *Plasmodium* parasites have also been confirmed in multiple non-native species in New Zealand [17–21], with the suggestion that some may be acting as reservoirs for spillover infection to native species [19]. The exact number of *Plasmodium* lineages (defined by unique sequences of parasite mitochondrial cytochrome *b* DNA) in New Zealand is unclear, but as many as 17 have been reported, with four generally accepted non-native lineage clusters [17,20,22]. Additional unresolved lineages have been reported, including some speculated to be native to New Zealand [23].

Although avian malaria has impacted both captive populations and wild individuals in New Zealand, whether or not wild populations have been impacted is unknown. Additionally, while studies exist that have investigated latitudinal patterns of infection in New Zealand [19,23], no such studies have explored elevational patterns. Previous research conducted in Nelson Lakes National Park (NLNP; South Island, New Zealand) indicates a general decline in bird abundance during the last 30 years [24], with greater declines below an apparent elevation threshold of around 1000 m. The authors suggested this is due to predation, with greater pressure at low elevation. However, it could also be that malaria, as a mosquito-borne disease, is involved [25]. Since mosquito densities are highly dependent on temperature, transmission rates also tend to be higher at low elevation [26].

We conducted a survey to identify patterns of avian malaria in wild birds in NLNP. The overall aim was to test the potential for avian malaria to have played a role in population-level declines in native birds at the study site. Since one of the components determining the rate at which a host population acquires infection (known as the force of infection; [27]) is infection prevalence (i.e. the proportion of infected individuals), the study was designed to test predictors of malaria prevalence in native bird populations. Specifically, we hypothesized that prevalence of infection is positively associated with a decrease in elevation, and higher in non-native than in native host species.

## Materials and Methods

### Study site and sampling

Sampling was conducted along the St. Arnaud Range in NLNP. This area of the St. Arnaud Range is included within the Rotoiti Nature Recovery Project (RNRP), one of six mainland ecological restoration projects (‘mainland islands’) in New Zealand [28]. These mainland islands are sites for various ecological restoration efforts and wildlife management. The RNRP hosts a diverse avifauna, including many threatened or declining species such as South Island robin (*Petroica australis*), kaka (*Nestor meridionalis*), and rifleman (*Acanthisitta chloris*). Additionally, the RNRP is being used for great spotted kiwi (*Apteryx haastii*) translocations. The St. Arnaud range consists of native southern beech (*Nothofagus* spp.) rainforest, ranging in elevation from 620 m to an abrupt tree line at approximately 1400 m. A domestic sheep pasture and the village of St. Arnaud (population approximately 500) border the St. Arnaud range at approximately 800 and 650 m in elevation, respectively.

Sampling took place at four elevations (650, 800, 1200, and 1400 m), located above and below the reported bird decline threshold elevation of 1000 m [24]. Native forest habitat was sampled at one site at each elevation; sampling was additionally undertaken at one site within the village (650 m), and one site on the boundary between the sheep pasture and forest (800 m). Samples were thus collected from six different sites at four elevations: 650 m (village: 41°48'16"S, 172°50'35"E; forest: 41°48'42"S, 172°51'3"E), 800 m (pasture edge: 41°48'33"S, 172°52'12"E; forest: 41°48'44"S, 172°52'5"E), 1200 m (forest: 41°48'15"E, 172°53'55"E) and 1400 m (forest: 41°49'22", 172°52'46").

Birds were captured by mist netting between sunrise (0500 hr) and an hour before sunset (2100 hr) during the warmer months for three consecutive years, 2012–13 (March-April), 2013–14 (November-March), and 2014–15 (January-April). Due to the various sampling locations and uneven capture rates, mist-netting effort was standardised by calculating the total number of birds caught per 100 net hours. All birds were banded with numbered metal leg bands to prevent re-sampling. After swabbing with 70% ethanol, 50–100 μl of blood was collected by capillary tubes via brachial venipuncture with 27 gauge needles, and stored unfrozen in a Queen’s lysis buffer [29] until processed for molecular analysis. Birds were sampled under Otago University Animal Ethics Approval 2/13 and New Zealand Department of Conservation permit 35873-FAU.

### Parasite detection

DNA was extracted from blood samples using a DNeasy Blood & Tissue Kit (QIAGEN, Valencia, California, USA) following a modified protocol [30]. Samples were screened for the presence of *Plasmodium* spp. (and *Haemoproteus* spp., a related parasite) using nested polymerase chain reaction (PCR) to amplify a 478 bp fragment of the mitochondrial cytochrome *b* gene following a method developed by our colleagues [31] with slight modifications [30]. Nested PCR products were purified with Exo-SAP (USB, Cleveland, OH, USA) and run on an ABI 3730xl DNA Analyser by the Genetics Analysis Service, University of Otago. *Plasmodium* lineages were identified by performing a BLAST search on the NCBI GenBank nucleotide database and the MalAvi database (Bensch *et al*., 2009).

### Data analysis

Due to low sample sizes at each of the higher elevation sites, the sampling sites were combined into two elevational groups (Egroup): “lower” (650 and 800 m) and “higher” elevation (1200 and 1400 m). Bird species were also placed into two different bird groups (Bgroup) by New Zealand classification: non-native and native. An assessment of a habitat type effect (habitat, i.e. forest, pasture edge or village) and sampling year effect (year) was also considered in the analysis.

Generalised linear models were used to explore the independent effects of the four variables on malaria prevalence. We used logistic regression to model malaria prevalence (infected = 1, not infected = 0) against four fixed factors (year, habitat, bird group, and elevation group). A model selection approach was used to identify the simplest best fitting model using Akaike’s Information Criterion (AIC) (Table 1), with the lowest AIC value indicating the best fitting model [32]. Bird age and sex were not included in this analysis due to a large number of missing values. Chi-square tests and two-tailed Fisher’s exact tests (if the frequency in one or more groups was less than five) were used to compare prevalence between malaria parasite lineages using VassarStats [33]. All other analyses were performed using the statistical software IBM SPSS Statistics version 20.0 (SPSS Inc. Chicago, IL, USA). A significance level of 0.05 was used for all analyses.

**Table 1:** Summary of backward model selection of Akaike’s information criterion (AIC) for regression models of four independent variables (Year, Habitat, Bgroup, and Egroup) against the binomial dependent variable of malaria infection (infected = 1, not infected = 0) of wild birds of Nelson Lakes National Park, New Zealand. ΔAIC = difference in AIC between the current model and the best model.

Model	K	Log-Likelihood	AIC	ΔAIC
1. Year+Habitat+Bgroup+Egroup	4	-18.57	**51.14**	**0**
2. Year+Habitat+Bgroup	3	-19.63	**51.27**	**0.13**
3. Habitat+Bgroup+Egroup	3	-26.96	63.92	12.78
4. Habitat+Bgroup	2	-28.23	64.46	13.32
5. Bgroup	1	-37.27	78.54	27.40
6. Year+Bgroup	2	-35.61	79.23	28.09
7. Bgroup+Egroup	2	-37.22	80.44	29.30
8. Year+Bgroup+Egroup	3	-35.61	81.23	30.09
9. Null	Intercept	-50.40	102.79	51.65
10. Egroup	1	-49.66	103.32	52.18
11. Habitat+Egroup	2	-47.79	103.58	52.44
12. Habitat	1	-49.14	104.27	53.13
13. Year+Habitat+Egroup	3	-46.80	105.61	54.47
14. Year	1	-49.85	105.71	54.57
15. Year+Habitat	2	-48.14	106.27	55.13
16. Year+Egroup	2	-49.20	106.39	55.25

Year = sampling year; Habitat = habitat type; Bgroup = non-native or native bird classification, Egroup = elevational grouping of sample sites; K = number of parameters.

## Results

### Data summary

**Fig 1:**
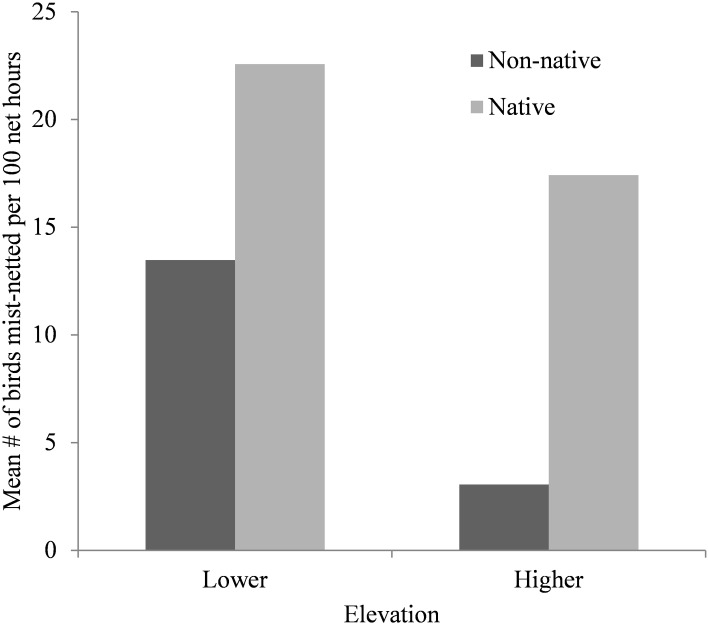
Standardised mist-net capture rates of wild birds for three consecutive years (2012–13 to 2014–15) at Nelson Lakes National Park, New Zealand. Lower elevation = 650 and 800 m; higher elevation = 1200 and 1400 m.

Blood samples were collected and analysed from 436 individual birds representing 15 species from 11 families (Table 2), with 369 samples collected from lower elevations (650 and 800m) and 67 samples from higher elevations (1200 and 1400m; Table 3). More non-native birds were caught at lower than higher elevation, whereas numbers for native birds was similar between elevations (Fig 1). These results were obtained by standardising the capture rates, based on the total number of net hours for lower and higher elevations calculated at 1024 and 327, respectively. *Plasmodium* spp. was detected in a total of 26 blood samples from three of six non-native species, and two of nine native species. No *Haemoproteus* parasites were detected in this study. An overall malaria prevalence of 6% (n = 436) was observed (Table 3).

**Table 2:** List of sampled avian host species and *Plasmodium* lineages identified at four elevational steps at Nelson Lakes National Park during the warmer months for three consecutive years from 2012–13 to 2014–15.

Family	Species	650 m	800 m	1200 m	1400 m	Total	*Plasmodium* lineage
**Non-native hosts**		**7/86 (8%)**	**12/52 (23%)**	**1/1 (100%)**	**1/9 (13%)**	**21/149 (14%)**	
Fringillidae	Goldfinch *Carduelis carduelis*	-	0/3	-	-	0/3 (0%)	
	Chaffinch *Fringilla coelebs*	0/8	0/23	-	0/1	0/32 (0%)	
Muscicapidae	Eurasian blackbird *Turdus merula*	4/11	8*/15	1/1	0/4	13/31 (42%)	LINN1 (10); LINN1/GRW06 (1)*; SYAT05 (2)
	Song thrush *Turdus philomelos*	0/1	4/9	-	1/1	5/11 (45%)	LINN1 (5)
Ploceidae	House sparrow *Passer domesticus*	3/61	-	-	-	3/61 (5%)	GRW06 (3)
Prunellidae	Dunnock *Prunella modularis*	0/5	0/3	-	0/3	0/11 (0%)	
**Native hosts**		**0/35 (0%)**	**6/196 (3%)**	**0/26 (0%)**	**0/31 (0%)**	**5/287 (2%)**	
Acanthizidae	Grey warbler *Gerygone igata*	0/1	0/2	-	0/1	0/4 (0%)	
Eopsaltriidae	Tomtit *Petroica macrocephala*	-	0/28	0/12	0/15	0/55 (0%)	
	South Island robin *Petroica australis*	0/3	1/4	0/1	-	1/8 (13%)	SYAT05 (1)^†^
Meliphagidae	Bellbird *Anthornis melanura*	0/4	0/44	0/5	0/10	0/63 (0%)	
	Tui *Prosthermadera novaeseelandiae*	0/3	0/3	-	0/1	0/7 (0%)	
Monarchidae	Fantail *Rhipidura fulginosa*	0/1	0/7	0/1	0/1	0/10 (0%)	
Pachycephalidae	Brown creeper *Mohoua novaeseelandiae*	-	-	-	0/2	0/2 (0%)	
Xeniciddae	Rifleman *Acanthisitta chloris*	-	-	0/7	-	0/7 (0%)	
Zosteropidae	Silvereye *Zosterops lateralis*	0/23	4/107	-	0/1	4/131 (3%)	SYAT05 (4)^†^
**Total**		**7/121 (6%)**	**18/248 (8%)**	**1/27 (4%)**	**1/40 (3%)**	**26/436 (6%)**	

Values indicate the number of infected individuals over the total number examined with prevalence in parentheses.

* Indicates a double infection.

^†^ Indicates a new lineage-host association.

**Table 3:** *Plasmodium* prevalence in wild birds of Nelson Lakes National Park, by year, elevation, and bird group.

	2012–13	2013–14	2014–15	Total
**Non-native hosts**	**10/57 (18%)**	**7/67 (10%)**	**4/22 (18%)**	**21/148 (14%)**
Lower elevation	9/56	6/59	4/21	19/138 (14%)
Higher elevation	1/1	1/8	0/1	2/10 (20%)
**Native hosts**	**3/124 (2%)**	**0/88 (0%)**	**2/78 (3%)**	**5/288 (2%)**
Lower elevation	3/98	0/63	2/72	5/231 (2%)
Higher elevation	0/26	0/25	0/6	0/57 (0%)
**Total**	**13/181 (7%)**	**7/155 (5%)**	**6/100 (6%)**	**26/436 (6%)**

Values indicate the number of infected individuals over the total number examined, with prevalence in parentheses.

### Model factors influencing malaria prevalence

**Fig 2:**
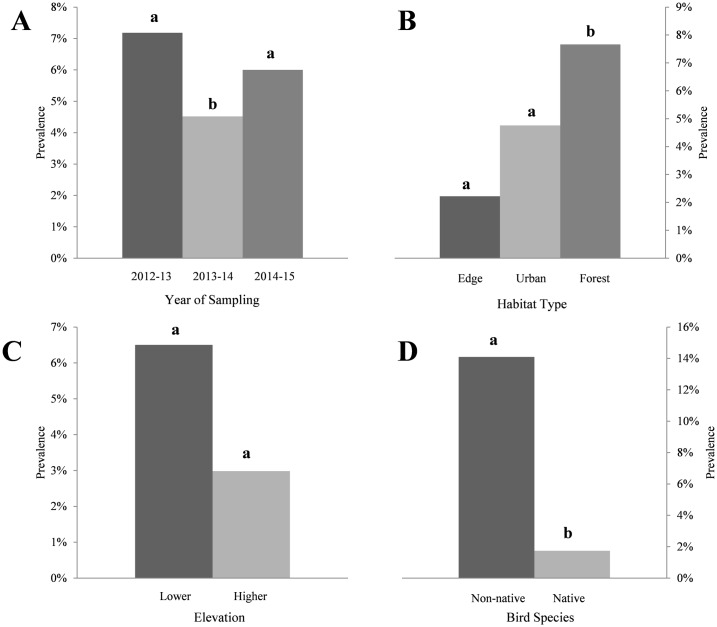
Summary of *Plasmodium* prevalence data at Nelson Lakes National Park, New Zealand. (A) Year of sampling, (B) habitat type, (C) elevational group, (D) non-native or native status of bird host. Columns within categorical groups with different letters differ (P < 0.05). Note the differences in scale.

Model selection resulted in two candidate models (ΔAIC < 2) that best explain the variation in malaria prevalence in this study (Table 1). Values from Model 1, containing all four factors, are used herein. Overall prevalence of infection varied by year (Wald χ^2^_2_ = 12.384, p = 0.002) (Table 4), with prevalence in 2012–13 being 7.2% (n = 181), 2013–14 being 4.5% (n = 155), and 2014–15 being 6.0% (n = 100) (Table 3). A significant difference was observed between years 2013–14 and 2014–15 (P = 0.008) (Fig 2). Prevalence of infection also varied by habitat type (Wald χ^2^_2_ = 22.987, P < 0.0001) (Table 4), with prevalence of 2.2% (n = 45), 4.8% (n = 105), and 7.7% (n = 261) for pasture edge, village, and forest habitats, respectively (Fig 2). A significant difference was observed between village and forest habitats (P = <0.0001) (Fig 2). Birds sampled at lower and higher elevations showed prevalence of 6.5% (n = 369) and 3.0% (n = 67), respectively (Table 3). Although the difference between elevations was not significant (Wald χ^2^_1_ = 1.761, P = 0.185), a trend was observed showing an inverse relationship between prevalence of infection and elevation (Fig 2).

**Table 4:** Factors predicting *Plasmodium* infection in wild birds of Nelson Lakes National Park, New Zealand.

Predictor	df	Wald Chi-Square	p-Value
Year	2	12.384	0.002
Habitat	2	22.987	< 0.001
Bird group	1	36.366	< 0.001
Elevation group	1	1.761	0.185

Year = sampling year; Habitat = habitat type; Bgroup = non-native or native bird classification; Egroup = elevational grouping of sample sites.

When bird species were grouped, parasite prevalence was significantly higher in non-native species (14.1%; n = 148) than in native species (1.7%; n = 288; Wald χ^2^_1_ = 36.366, P < 0.0001) (Table 3; Fig 2). Of the five bird species that were positive for malaria parasites, Eurasian blackbird (*Turdus merula*) and song thrush (*T*. *philomelos*) showed the highest overall parasite prevalence; 42% (n = 31; CI 26–59) and 45% (n = 11; CI 21–72), respectively (Table 2). Parasite prevalence for house sparrow (*Passer domesticus*), South Island robin, and silvereye (*Zosterops lateralis*) was 5% (n = 60; CI 2–14), 13% (n = 8; CI 2–47), and 3% (n = 131; CI 1–8), respectively.

### Malaria lineage identification

**Fig 3:**
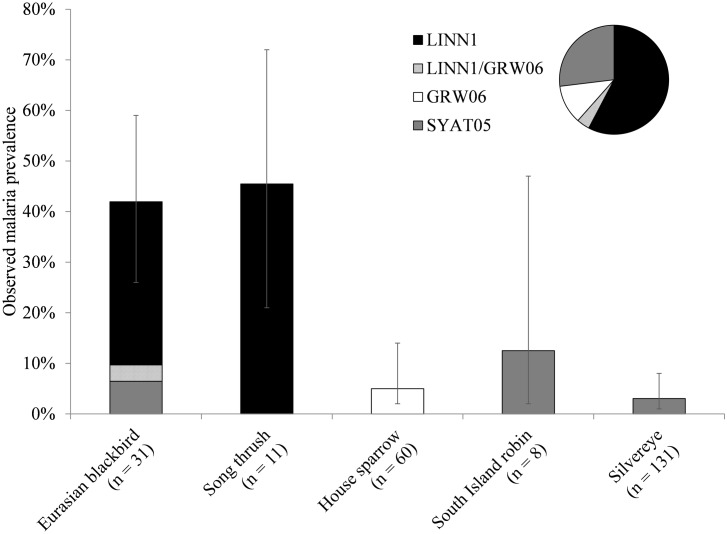
Observed prevalence of *Plasmodium* lineages in five avian species at Nelson Lakes National Park, New Zealand. Non-native host species with parasite infections are Eurasian blackbird (*Turdus merula*), song thrush (*Turdus philomelos*), and house sparrow (*Passer domesticus*), with no infections in three other species. Native species with parasite infections are South Island robin (*Petroica australis*) and silvereye (*Zosterops lateralis*), with no infections in seven other species. The error bars represent the lower and upper bounds of the 95% confidence interval. The pie chart shows relative proportion of positive infections by each lineage (n = 26).

**Fig 4:**
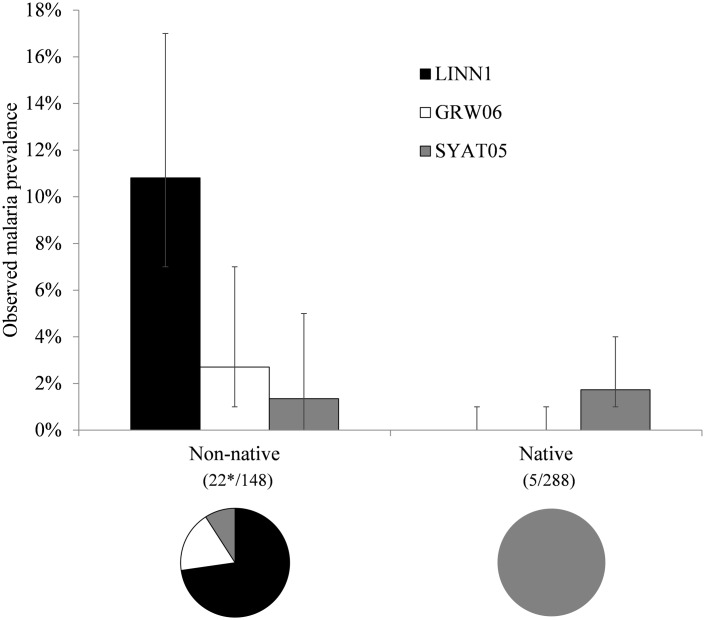
Observed prevalence of *Plasmodium* lineages in non-native and native host species in Nelson Lakes National Park, New Zealand. The error bars represent the lower and upper bounds of the 95% confidence interval. The pie charts show relative proportion of positive infections by each lineage within bird groups. The numbers in parentheses represent number of individuals infected and screened respectively for each group. *Note, one double infection (lineages LINN1 and GRW06) was found in one non-native blackbird (*Turdus merula*) individual and lineages are displayed here as independent observations.

All positive samples were successfully sequenced and three avian malaria lineages were identified (Table 2; Fig 3). Sequences showed a 99–100% similarity to published sequences in GenBank and MalAvi (except for the lineage identified from the South Island robin, which showed a 95% similarity using forward sequence only). These were *Plasmodium* sp. lineage LINN1 (GenBank GQ471953, MalAvi LINN1), *P*. *(Huffia) elongatum* lineage GRW06 (GenBank DQ368381, MalAvi GRW06), and *P*. *(Novyella) vaughani* lineage SYAT05 (GenBank DQ847271, MalAvi SYAT05). Lineage LINN1 was the only lineage found at the higher elevation sites, while lineage SYAT05 was the only lineage found in native bird species. One double infection (lineages LINN1 and GRW06) was found in one blackbird individual at 800 m. Prevalence was significantly higher in non-native hosts than in native hosts for both lineages LINN1 (χ^2^ = 29.35, d.f. = 1, P < 0.0001) and GRW06 (Fisher's exact test, P = 0.0129) (Fig 4), while there was no such difference for lineage SYAT05 (Fisher's exact test, P = 0.5654).

## Discussion

### Epizootiology

This study was the first investigation of avian malaria presence in non-native and native birds across an elevational gradient in New Zealand. The results revealed differences in the elevational distribution and species composition of the host-parasite community studied. Analysis of the data also revealed prevalence varied by year and habitat, with higher *Plasmodium* prevalence in native forest habitats. The difference between years may be correlated with a difference in mosquito emergence due to temperature or rainfall variation among years; however more research is needed to determine this. The difference in prevalence between habitats, with a higher prevalence observed in forest habitat, could be due to the different number and type of host species sampled in each habitat type. Given that the observed prevalence was higher in non-native versus native birds, it is possible that the non-native versus native status could be driving differences observed by habitat as the bird species sampled are not randomly distributed across habitat types. The difference in prevalence between habitats may also be related to vector density, with *Culex pervigilans* being the only suspected avian malaria vector reported at the study site [34]. Although this mosquito species uses a wide range of breeding sites, it has been reported as more common in natural breeding sites compared to artificial containers [35].

As hypothesised, we observed higher malaria prevalence in non-native species than in native species (Fig 2). These findings are similar to those from previous surveys conducted in New Zealand [17,19–21]. Although our second hypothesis did not hold up in a statistically significant manner (there was only a trend of malaria prevalence decreasing with increasing elevation), our findings with regards to prevalence in native versus non-native birds, combined with there being more non-native birds at lower elevations at our field site (Fig 1) and elsewhere in NLNP [24], mean that there were more infected birds (from which malaria could be transmitted) at lower elevations. In addition, a greater abundance of mosquitoes exists at lower elevations [34], increasing the potential for vector-host encounters. As a result, the force of malaria infection will indeed have been greater at lower than higher elevation. Although the higher elevation sites appear to be mosquito-free [34], two infected birds were sampled from these sites. One possible explanation is that transmission occurred at the same location as sampling took place, due to mosquitoes migrating from lower elevations under optimal temperature conditions [26]. However it is more likely that transmission occurred at lower elevations, especially since some host species, such as blackbirds and song thrush, are capable of moving along an elevational gradient throughout the year in New Zealand [36]. Since both the malaria parasite and reservoir host have been detected at elevations as high as the tree line, the presence of a competent mosquito vector may be the only missing component of the malaria transmission cycle at these higher elevations. This may have important implications for species or populations occurring in greater numbers at higher elevations such as the brown creeper (*Mohoua novaeseelandiae*), due to the threat of climate change and with it the potential upward elevational movement by mosquitoes [13].

The observed difference in the infection prevalence of native versus non-native birds could be determined by drivers at the vector, pathogen, or host level. Possible factors include rates of transmission, parasite fitness, and host susceptibility [11,37,38]. If differences in transmission rates caused the observed prevalence patterns, we would expect native birds to be exposed to mosquitoes less often than non-native birds. *Culex pervigilans* is a native mosquito, and therefore existed before the introduction of the current non-native avifauna, in an ecosystem with very few native terrestrial mammals (two genera of bats only; [39]). Thus it is likely that historically, native birds represented the majority of blood meals taken; however it is possible that due to the millions of years of co-existing, native birds could have developed avoidance strategies to *C*. *pervigilans*, leaving non-native birds more susceptible to this particular mosquito species. We do know however, that some transmission is occurring in native birds, by the evidence of malaria positive individuals. Additionally, *C*. *pervigilans* is the most prevalent and widespread mosquito in New Zealand [35], suggesting a wide host range and providing further support against the possibility of lower transmission rates in native birds. Another possible driver of the observed patterns in prevalence may be native bird species being less suitable hosts for malaria parasites than the non-native species found in the area, and thus affecting the survival rate of the parasite within hosts. This is unlikely, however, since *Plasmodium* parasites have a broad host range worldwide [40–42], and some show a high level of evolutionarily stable host-switching [8]. Alternatively, it is also possible that lower parasite prevalence in native birds could be contributed to greater susceptibility to infections due to weakened immune systems, resulting in native birds actually being more resistant, rather than the other way round. Of note, all the host species sampled in this study are non-migratory, thus minimizing the potential for parasite introductions to individuals while outside of New Zealand.

Although we are not ruling out the possibility that either transmission efficiency or parasite fitness may explain the host species patterns observed, it is more likely they are related to differential parasite virulence. The increased mortality of native birds due to exotic strains of malaria (as was observed in Hawaii; [7,43]) could account for the lower observed prevalence in these species. In addition, there exists an inherent sampling bias with mist-netting (the sampling methodology used), as sick or weak birds may not be caught due to inactivity. Since only birds below a certain level of parasitemia tend to be active [5], more native birds than non-native birds being weakened by infection may also contribute to the observed pattern. Also, birds with higher parasitemia levels are at a greater risk of predation [44], further biasing reported malaria prevalence in wild populations.

### Malaria patterns by lineage

The three malaria lineages detected in this study, *Plasmodium (Huffia) elongatum* lineage GRW06, *P*. sp. lineage LINN1, and *P*. *(Novyella) vaughani* lineage SYAT05, belong to separate non-native lineage clusters. All are globally widespread and have previously been reported in both native and non-native bird species in New Zealand [20,22]. Lineage GRW06 is considered to have the widest host range in New Zealand [22]. Lineage LINN1 has only been reported in native species in extremely low numbers [17,20,21,45], two instances of which involved deaths of great spotted kiwi due to malaria infection [17,46].This could have implications at NLNP where currently exists a population of approximately 25 great spotted kiwi, with documented cases of successful breeding [47]. Lineage SYAT05 is commonly reported in studies conducted in the North Island, however we found no references in the literature from the South Island prior to this study. To the authors’ knowledge, this is the first study to show the presence of lineage SYAT05 infection in silvereye and South Island robin, further highlighting the gaps in knowledge of this system in New Zealand, especially in the South Island.

A pattern of higher prevalence in non-native birds than in native birds was observed for lineages LINN1 and GRW06, but not for lineage SYAT05 (Fig 4). These contrasting patterns are likely not a direct result of parasite spillover (even though this may be occurring at our field site), as we would expect to observe the same proportion of lineages in non-native and native hosts. This uneven distribution is also unlikely to be due to the local distribution of *Plasmodium* parasites at the field site in NLNP, since infected *Turdus* spp. individuals were caught at five of the six mist-netting locations. So why does prevalence vary dramatically within the same host population for only two of the three lineages? The most likely explanation is that any negative impacts from *Plasmodium* parasites on native New Zealand birds are dependent on the specific *Plasmodium* lineage parasitising the host, with some lineages (in this case lineage LINN1) having more of an impact than others. However, to further explore this hypothesis, experimental infection trials would be needed to assess host susceptibility to the various strains found throughout New Zealand. Based on the prevalence data from this study, it is reasonable to assume that if malaria is affecting native birds in NLNP at a population level, then lineage LINN1 is likely to be involved.

### Key reservoir hosts

Given the generally low avian malaria prevalence reported in native birds in New Zealand (with only two or three exceptions; [20,21]), it is indeed most likely that non-native species act as the primary reservoirs for avian malaria in New Zealand [19]. Based on data from the current study, we suggest that the *Turdus* spp. (especially the Eurasian blackbird) may be key reservoir hosts for malaria parasites in NLNP. The blackbird is abundant throughout both the North and South Island and is a known reservoir of *Plasmodium* parasites in other countries, specifically implicated as a key reservoir host [48]. In this study, both the blackbird and song thrush showed a significantly higher prevalence than any other species (Fig 3), and were caught in all habitat types at all elevations. *Turdus* spp. individuals move frequently between habitat types during the breeding season [49] and can penetrate deep into New Zealand forests, while most other non-native bird species do not [50]. Similar patterns of malaria infection have been found in other areas of New Zealand, with *Turdus* species often having the highest prevalence and being caught in more habitat types than other non-native species [17,19–21]. Identifying potential key reservoir hosts in a disease system helps to better understand the factors driving transmission.

## Conclusion

In conclusion, our results support the potential for a higher force of avian malaria infection at the lower elevations of our field site. We report an overall prevalence of 6% (n = 436) for this study, which is slightly lower than previous records from the northern half of the South Island of New Zealand and likely due to the higher elevation at which sampling took place [19,23]. We also report an overall higher prevalence of malaria in non-native versus native birds living in a shared space, suggesting the possibility of a differential impact on host species that show dissimilar reservoir competence. Of note, no parasite was detected in the five native bird species (n = 137) undergoing decline in NLNP over the past 30 years, yet a high prevalence was observed in blackbirds, a species not reported as declining [24]. We suggest that despite reports of low malaria prevalence in native hosts, transmission may still be occurring, however with lower detection due to potential impacts of infection. Our findings provide further support to the hypothesis that disease, in addition to predation and competition, could be playing a role in observed population declines at this site [25].

We argue that one of greatest knowledge gaps regarding avian malaria in New Zealand is the lack of information on host susceptibility to infection, information that could be obtained through experimental infection studies. If the native avifauna is impacted by avian malaria at a population scale, and a differential impact on host species is occurring due to specific *Plasmodium* lineages, it would carry with it a whole new complexity to the conservation of native wildlife in New Zealand. Supporting evidence for such disease impacts will enable appropriate management to be developed and put in place, while a lack of evidence will enable resources to remain focused on the other issues facing New Zealand’s native species (e.g. through habitat restoration, pest control, translocations and genetic management). In the case of the former, providing managers with more specific information of any potential disease impacts on native bird populations could help avoid costly or less efficient “blanket” conservation and management efforts.
